# Establishing best-practice statements for post-custody community transition: Insights from a modified Delphi study

**DOI:** 10.1371/journal.pone.0323118

**Published:** 2025-05-08

**Authors:** Tazeen Majeed, Jo Taylor, Erica Breuer, Smriti Nepal, Marc Remond, Luke Grant, Stephen Hampton, Elizabeth Sullivan

**Affiliations:** 1 School of Medicine and Public Health, College of Health, Medicine and Wellbeing, University of Newcastle, New South Wales, Australia; 2 The Poche Centre for Indigenous Health, Faculty of Medicine and Health. The University of Sydney, New South Wales, Australia; 3 College of Health, Medicine and Wellbeing, University of Newcastle, New South Wales, Australia; 4 Corrective Services, New South Wales, Australia; 5 Primary Care Directorate, Justice Health and Forensic Mental Health Network, New South Wales, Australia; University of North Carolina at Chapel Hill, UNITED STATES OF AMERICA

## Abstract

**Introduction:**

The transition from custody to community is a critical juncture for people exiting custody, necessitating substantial support. However, the programs and services providing this support are often fragmented and lack a unified approach in terms of terminology, design, resourcing, timing, delivery, and monitoring and evaluation. This study sought to establish an agreed terminology and promote a broader consensus on best practices relating to these programs and services.

**Methods:**

We used a two-phased Delphi methodology to provide a structured, formal, and iterative process for gathering and refining the opinions of subject experts, knowledge holders, and people with lived experiences. Using purposive sampling, 160 national and international participants were invited to participate. Comprehensive study protocol has already been published elsewhere.

**Results:**

This modified two-phased Delphi study delivers a set of 40 best-practice statements that can be adapted to the individual needs and contexts of different programs and services. These best-practice statements clarify several key themes, including language and terminology, program aims and outcomes, best-practice principles, the significance of an interconnected phased approach, timing related to services and programs, responsibility for funding and coordination of programs, and monitoring and evaluation. This study introduces an umbrella term, ‘Transitional and Post-Release Support Programs (TPSP)’, to describe programs and services for people exiting custody and provides best-practice statements that can enhance access, documentation, monitoring, and evaluation processes.

**Conclusion:**

This study underscores the importance of a value-based approach in TPSPs to foster purposive program design and a human rights-aligned approach to service provision for people exiting custody. The use of umbrella term and the best-practice statements will promote the use of humane, dignified language, a streamlined, timely, structured, and collaborative discourse, and a more cohesive approach to service provision for people exiting custody.

**What is already known on this topic**

-  People exiting correctional facilities face significant challenges in securing their human rights despite existing standards like the Universal Declaration of Human Rights and the Nelson Mandela Rules.
-  Existing programs and services for people transitioning from custody are often disjointed, vary greatly in policies and practices across the world, and lack a unified approach in terms of design, resourcing, and evaluation.

**What this study adds**

-  We introduce the umbrella term “Transitional and Post-Release Support Programs (TPSP)” to describe these programs and services and develop 40 best-practice statements to enhance the design, delivery, and evaluation of TPSPs.

**How this study might affect research, practice of policy**

-  The findings highlight three pivotal opportunities: adopting a common, destigmatizing language for TPSPs to foster international collaboration and a common ground for stakeholder engagement; developing an evidence-informed framework for designing, monitoring, and evaluating TPSPs; and creating efficient, human rights-based programs to enhance individuals’ outcomes and community survival as they transition out of correctional facilities.

## Introduction

Securing the human rights of people experiencing incarceration has always been challenging. Despite the presence of various human rights standards such as the Universal Declaration of Human Rights (1948), Standard Minimum Rules for the Treatment of Prisoners (1955) and the Nelson Mandela Rules (2015), [1] people continue to face inequities and inequalities, before, during and after incarceration. The fourth Nelson Mandela Rule specifically states that imprisonment should aim to promote desistance by facilitating the reintegration of people into self-supporting, law-abiding life on release [2]. The rule states that to achieve this, correctional facilities should offer education, vocational training, and diverse forms of assistance tailored to the individual needs of the people in custody. However, not all correctional facilities offer this support in accordance with Human Rights guidelines [3].

The transition from custody to the community at the completion of their sentence is a critical juncture where people exiting correctional facilities seek to rebuild their future. They require substantial support to successfully navigate this juncture and to avoid reoffending and potentially returning to custody [4–7]. Central to this process are programs and services supporting the transition to, and reintegration into, the community. Globally, many correctional facilities offer programs and services to people in custody and during their post-release time to aid their transition to the community. However, the underlying policies, aims and objectives, practices and provision of these programs and services vary widely. For example, high-income Nordic nations place a strong emphasis on rehabilitation through all-inclusive, individually-tailored programs that incorporate education, career training, and mental health support [8]. In contrast, low-income countries are constrained by overpopulated custodial settings and limited funding, resulting in ineffective rehabilitation initiatives [9]. The United States offers many re-entry programs, covering multiple domains, including educational, employment readiness and substance use programs [10]. However, their availability and quality vary significantly between federal and state correction systems. Programs and services offered in Australia also vary significantly across jurisdictions due to differing legislative mandates and correctional service structures [11]. Even when such programs and services are available, one common problem reported is that they are not always gender-specific [4,12], culturally appropriate [13,14], or relevant for people with diverse needs.

There is increasing acknowledgement of the importance of researchers implementing a human rights perspective approach that intentionally uses language that promotes de-stigmatization of people in custody. More specifically, this approach uses language that respects and embraces the humanity and dignity of the incarcerated people, and their families, and avoids labelling people or their experiences [15–18], For example, using terms like ‘patient’ instead of ‘prisoner-patient’ helps to recognise their humanity and reduce stigma [15]. Inappropriate labelling can reinforce stigma and act as a driving factor for stereotyping and discriminatory attitudes, which in turn can shape people’s views about this already marginalised group, thereby impacting their future, health and wellbeing [15]. Additionally, language, when not used appropriately, creates a paradigm that deprives people of their complex identities. Despite this, both internationally and in Australia, there is a lack of consensus around appropriate language and terminology to be used in defining and describing programs and services for people exiting custody. This lack of consensus and uniformity observed around language and terminology also extends to a lack of consensus in relation to the design, implementation, monitoring and evaluation of such programs and services.

This creates a complex and fragmented landscape that challenges the very essence of successful reintegration. This is evident in the variability of the language employed to describe programs and services, that leads to confusion and misalignment among stakeholders. For example, in some jurisdictions, the focus might be on “re-entry programs,” emphasising the process of returning to society, while in others, “rehabilitation programs” may be the preferred term, reflecting an emphasis on personal change and growth. These semantic differences not only complicate program advocacy, engagement and national and international benchmarking but also obscure the true goals and outcomes of these initiatives.

In addition to the variety of terminology used to describe such programs and services, there is variability and inconsistency in the design and delivery of these, including differences in aims, intended outcomes, components, and timing of program commencement. Illustrative of this is that some services or programs focus more on providing housing support while others emphasise personal skill development. Some jurisdictions offer pre-release programs, while others focus on post-release support. The timing of such interventions can profoundly affect an individual’s prospects for successful reintegration [19], yet there is no consensus as to when is the most effective time to begin and end interventions [20].

The lack of continuity in services is another major concern. While individuals transitioning from custody may be able to access an array of services, the lack of coordination and continuity between these can undermine their effectiveness [19,21]. Moreover, disparities in access to different services such as housing, employment, mental health support, and substance abuse treatment only serve to complicate the reintegration process, further emphasising the need for comprehensive and coordinated programs [19,21,22].

These challenges point to the need for a comprehensive, harmonised set of recommendations to guide the use of language and terminology and the development and implementation of these services and programs, which have the power to change future social behaviours. Thus, the aim of this study was to: i) establish an agreed terminology to define and describe the programs and services offered to people transitioning out of custody and ii) generate wider consensus on best-practice statements relating to the aims, intended outcomes, components, attributes, timing, responsibility for funding and provision, and the monitoring and evaluation of these programs and services.

## Methods

The Delphi method is a structured tool, developed as a systematic, methodical, participatory forecasting process relying on a panel of experts. Using a succession of questions with controlled feedback in between, it is frequently utilised to reach a consensus on complicated subjects [23,24]. In the traditional Delphi approach, experts answer questions anonymously throughout several rounds of the process, with a facilitator summarising their answers and providing feedback at the end of each cycle. With each new round, experts update their responses in light of the facilitator’s feedback, progressively coming to a consensus [23,24]. Given the lack of consensus on the best-practice guidelines for services and programs for people exiting custody, a Delphi study was considered an appropriate research method [20]. We modified the traditional Delphi method and incorporated additional elements such as an evidence-based preparatory phase to develop best-practice statements, purposive and snowball sampling that included subject experts, stakeholders from service providers and people with lived experiences, and an online meeting with participants after the round 1 survey [20]. This modification better suited the research aims, provided a formal, iterative process to solicit and distil the opinions of subject experts, knowledge holders and people with lived experiences using a structured framework and allowed for more dynamic and immediate feedback, enhancing the overall quality of the consensus [25–28]. A comprehensive protocol for this study detailing the methodology has been previously published [20]. Below, we describe the most pertinent details of the methodology that are reported using the ACCORD checklist (See Supporting Information S3) [29].

### Sample

We adopted the definition of “Delphi experts” previously described by Blaschke et al. [30] and aimed to invite a panel of national and international participants who possessed both knowledge and experience representative of their capacity to articulate informed opinions and provide relevant input about the topic. The recruitment target for the initial phase of this study (Round 1, survey 1 – see details below) was set at 65 participants. Previous research recommends a minimum panel size of 15–20 participants to ensure appropriate contributions in a Delphi setting [31]. Considering the multisectoral nature and complexity of the programs and services offered to people transitioning out of custody, we aimed to include participants from all relevant groups, including First Nations people, people of Culturally And Linguistically Diverse (CALD) backgrounds, people with lived experience of incarceration and people living with a disability. Our target sample size allowed for the input of diverse views while accounting for expected attrition. To ensure that our target sample was met, we invited 160 national and international participants, including subject experts, key stakeholders from various service providers including, Community and Justice Services, and Not-for-Profits, First Nations stakeholders, those with lived experiences, researchers and healthcare providers (referred to as study participants from hereon), to engage in the consensus-building phase of this study.

### Recruitment

We primarily used purposive sampling to recruit the study participants. We approached subject experts and researchers known to the research team through their work in the area and asked key stakeholders at Corrective Services New South Wales and the Justice Health and Forensic Mental Health Network to recommend potential participants. We also identified names of potential participants from websites of Australian and international agencies and advocacy organisations based on their expertise in the subject area. To ensure a broad range of perspectives and opinions, we also used snowball sampling̶ asking participants to nominate other potential participants for inclusion in the study. We also included people with lived experience of incarceration, who had previously disclosed their experience in the public domain and were utilizing their experiences to work as advocates, academic researchers, or employees of Non-Government Organisations or governmental organisations. These individuals were recruited through a targeted approach, where we identified potential participants based on their public disclosures and professional roles. Invitations were extended to those who met the above criteria, ensuring that their insights and perspectives were represented in the study.

Once potential participants were identified, we emailed them either directly or using the Research Electronic Data Capture application (REDCap) [32] to invite them to participate in the study. Email invitations contained a soft copy of the Participant Information Sheet outlining the purpose of the study, methodology, potential risks and benefits of participation, anticipated study outcomes, the expected time contribution of participation, and a link to the online survey.

### Ethics

Participation was entirely voluntary with a written online consent form sent to participants with their invitation and also embedded at the start of the survey questionnaire. There were no incentives, and participants could withdraw at any time without any consequences. Those who agreed to participate and completed the consent process were included as study participants of the modified Delphi consensus-building process*.* Principles of confidentiality, privacy and anonymity of nominated participants were maintained throughout this process. Ethical approval has been received from the Justice Health Human Research Ethics Committee (JH File No.G217/16), Corrective Services Ethics Committee (CSEC; G217/16) and the Aboriginal Health & Medical Research Council (AH&MRC; HREC 1187/16). Ethics ratification was granted by the University of Newcastle HREC via expedited review (H-2022–0039).

### Modified two-phased Delphi process

**Fig 1:**
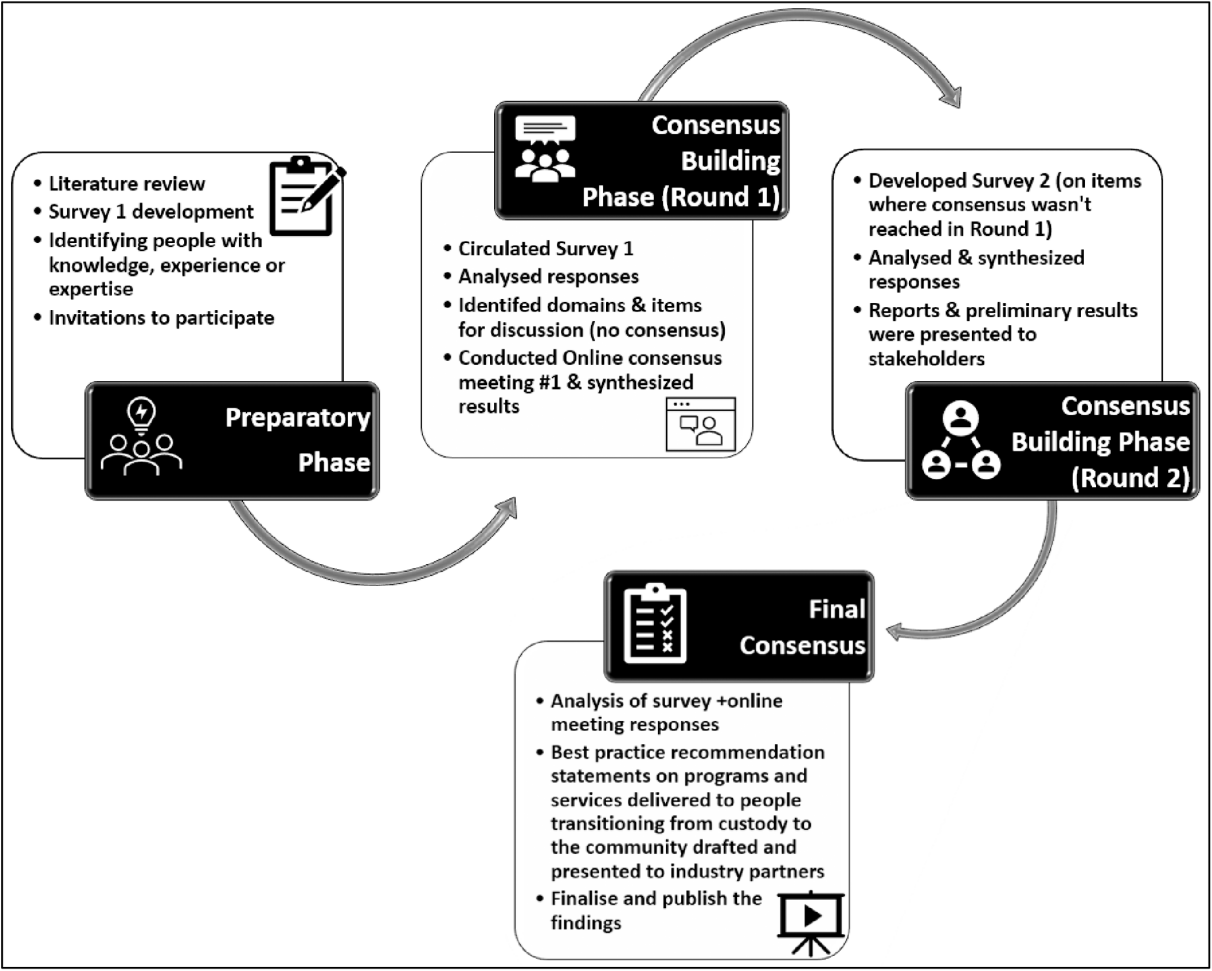
The modified Delphi process.

The Delphi was conducted in two phases – the preparatory phase and the consensus-building phase (see Fig 1):

i) ***The preparatory phase to develop candidate best-practice statements*** – this phase involved a systematic search and qualitative synthesis of published literature and online sources focused on programs and services for people exiting custody in order to develop a set of potential best-practice statements for these programs and services. Sources relating to all correctional facilities, including prisons, jails, detention centres etc. were included in this phase to ensure comprehensive coverage of incarceration settings. Full details of this search have been published elsewehere [20]. The potential best-practice statements developed through this process were allocated to one of six pre-defined domains: Definition and nomenclature, Aims and intended outcomes, Components and attributes, Timing, Responsibility and Monitoring and evaluation (See summary in Appendix, Fig a). Response items pertaining to each best-practice statement were developed for online Survey 1 using the REDCap [32–34]. The research team and a small sample of experts from community and justice services reviewed and piloted these response items several times before finalising them.
ii) ***The consensus-building phase*** was conducted over two rounds:
a) *Round 1 online survey* - participants completed an online survey of 298 response items pertaining to 40 best-practice statements. See Supporting Information S1 - ROUND 1, SURVEY 1 Questionnaire for details. Depending on the type of response item, participants were asked to rate (five-point Likert scale: Strongly Agree, Agree, Neither Agree nor Disagree, Disagree and Strongly Disagree) or rank (Essential, High Priority, Medium Priority, Low Priority, or Not a Priority) the response item, or choose a preferred response from a list of potential responses, or provide a free text response. Survey 1 also collected demographic information, including participant age, sex, current work and primary role, and experience. Consensus (positive or negative) was defined as > 80% agreement by participants on a response [27,35]. That is, for each statement and the subsequent items in the questionnaire, we defined “positive consensus” as occurring if > 80% of respondents indicated that they ‘Strongly Agree’ or ‘Agree’, or that they believe that a factor is ‘Essential’ or ‘High Priority’. We defined “negative consensus” as occurring if > 80% of respondents indicated that they ‘Disagree’ or ‘Strongly Disagree’ or that they believe that a factor is ‘Low Priority’ or ‘Not a Priority.” For statements that only allowed for a Yes/No response, “positive consensus” was defined as >80% of participants selecting ‘Yes’ while “negative consensus” was defined as > 80% of respondents selecting ‘No’.

Once the survey was completed, participant responses for each item were analysed. Statements that reached ‘positive consensus’ were included in the final best-practice statement, while those that reached ‘negative consensus’ were removed from the study. Statements not achieving positive or negative consensus were flagged as items for discussion at the online meeting.

*Round 1 online meeting* – A meeting was convened to present an update to survey participants about the results of the first round of the Delphi survey. Details on where consensus was or wasn’t reached were presented to all participants. In addition, a facilitated discussion guided by a chair (TM) and a co-chair (EB) was undertaken. This facilitation supported and enabled participants to review statements where consensus was not reached during survey 1 and to understand how to adapt these statements for participants to assess during a second round of the Delphi survey. Statements that had not reached positive or negative consensus during Round 1, or merged or modified versions of these statements that were agreed to during the online meeting, were included as survey items in Round 2, online questionnaire 2, as detailed below.

b) *Round 2 online survey* – The questionnaire was designed to gauge participant agreement on 12 potential best-practice statements that were generated based on Round 1 results. Statements in this round comprised 18 items for which consensus was not achieved during Round 1. See Supporting Information S2 - ROUND 2, SURVEY 2 Questionnaire for details.

### Data collection

Data collection for the Delphi process was conducted online over a three-month period (6^th^ October to 13^th^ December 2022) to provide sufficient time to gather data, aggregate and analyse responses, and build surveys step-wise (see timeline in Appendix 1, Fig b). A link to each survey was distributed via email to all participants with subsequent weekly reminder emails to increase the response rate.

### Analysis

We calculated descriptive statistics in STATA [36] in respect to responses for each best-practice statement rated by participants during Survey 1 and Survey 2.

Free text responses provided by participants during Survey 1 and participant comments and responses during the online meeting were integrated with the previously defined statements to produce new statements that were presented to participants in Survey 2. This data was analysed to generate clear and inclusive statements while maintaining the original purpose and meaning.

### Consensus-building Phase Round 1, Online Meeting 1

A simple approach to reflexive thematic analysis was undertaken using Nvivo [37] for coding of qualitative data collected during the online meeting [38]. We adopted a flexible and predominantly inductive approach to produce codes that were reflective of the content of the data, free from any preconceived theory or conceptual framework. Therefore, data was ‘open-coded’ to best represent meaning as communicated by the participants [39]. However, a degree of deductive analysis was employed to ensure that coding contributed to domains that were meaningful to the Delphi aims. The detailed methodology and consensus procedures are outlined in the ACCORD checklist (see Supporting Information - S3).

## Results

Of the 160 potential participants invited to participate in Round 1, Survey 1, 84 (response rate = 53%) consented to participate, with 61 participants submitting completed surveys and 23 submitting partially completed surveys. Approximately 93% of participants were from Australia, with the remaining participants from New Zealand (n = 3), United Kingdom (n = 2) and United States (n = 1). Appendix 1, Table 1 presents the demographic characteristics of participants in both Rounds.

**Table 1:** Demographic characteristics of Delphi participants.

	Round 1, Survey 1 (%) (N = 84)	Round 1, Online meeting (N = 28)	Round 1, Survey 2 (N = 35)
Geographic background			
Australia	n = 78	Not collected due to privacy considerations	n = 33
New Zealand	n = 3		n = 1
United Kingdom	n = 2		
United States	n = 1		n = 1
Gender			
Male	28.6		38.0
Female	70.0		59.0
Non-binary	0.0		0.0
Use a different term	0.0		0.0
Prefer not to say	1.4		3.9
First Nation People	11.4		0.0
Prefer not to say	2.9		3.0
People of Culturally and Linguistically Diverse (CALD) backgrounds	18.6		24.0
People with a disability	2.9		3.0
People with lived experience	7.2		5.0
Participated/been a part of program/service for people exiting custody (includes family members/ friends)	7.2		0.0
Current organisation where participants work			
State or federal government	40.0		46.0
Local health district	4.0		3.0
Aboriginal Community Controlled Health Organisations (ACCHO)/ Aboriginal and Torres Strait Islander community-controlled service	8.0		8.0
Pre or post service providers	2.0		3.0
Not for profit Research institute/ facility	13.0 24.0		15.0 20.0

***Round 1, Survey 1:*** Based on the criteria for measuring consensus outlined above, we reached consensus on 30 (75%) statements across five domains (Definition, Aims and intended outcomes, Components and attributes, Timing, Responsibility and Monitoring and Evaluation). There was no consensus on ‘Preferred term or nomenclature’ and ‘timing’ of commencement and continuance.

The majority of participants (61%) selected one of the following three options as their preferred term to describer services and programs offered to people exiting custody: ‘Transitional Support Program’ (33.3%), ‘Post-release Program’ (14.3%) and ‘Throughcare Program’ (13.1%). Given the lack of agreement on terminology, it was clear that this topic needed further participant discussion to reach consensus. There was also a lack of consensus around some key statements for best-practice definitions, attributes, and the responsibility for funding, provision and coordination. These statements and related items were flagged for discussion in the online meeting.

***Round 1, Online meeting:*** This was attended by 28 national and international participants (including three facilitators). Discussion during this meeting was focused on achieving agreement on a preferred term for programs and services offered to people as they prepare to exit correctional facilities. During the meeting, the panel discussed that even though there are a variety of programs and services offered to people as they transition from custody into the community, there was no agreed term that encompassed these programs and services. The panel further highlighted that these programs and services have fragmented funding, that they have differing aims, and that many sit at the intersection between health-related services and other programs. The panel agreed that reaching a consensus on an agreed ‘umbrella term’ to encompass programs and services for people as they transition through the correctional environment would empower and enable advocates, researchers, institutions, and other stakeholders to influence policy, secure funding, and measure and evaluate programs and services by introducing consistency across all sectors. Furthermore, there was a consensus within the panel that the terms ‘Transitional’ and ‘Post-release’ were preferred choices to be incorporated into this agreed ‘umbrella term’.

With respect to timing of service delivery, the majority of participants agreed that programs and services should start while people are incarcerated; however, there was no consensus on the exact commencement date and for how long they should be continued after release into the community. Therefore, modified statements and response items pertaining to this issue were included in Round 2, Survey 2.

A key theme that emerged from the online meeting was the importance of identifying key best-practice principles for programs and services offered to people as they transition from custody into the community. The panel agreed that these best-practice principles should encompass the fundamental and underpinning core values that inform the essential features of the programs and services.

As a result of feedback during the Consensus-building Phase Round 1, two domains and the relevant statements were revised. These were:

- The theme ‘definition and nomenclature’ from Round 1, Survey 1 was revised to ‘best-practice terminology’, and a list of eight terms was generated for inclusion in Round 2, Survey 2.
- A question on ‘agreed umbrella term’ was introduced.
- The theme ‘components and attributes’ from Round 1, Survey 1 was revised to ‘best-practice principles,’ and a list of eight core values was identified for inclusion in Round 2, Survey 2.

***Round 2, Survey 2:*** 77 participants from Survey 1 who had consented to take part in Survey 2 were invited. The response rate was 45%, with 34 participants completing the full survey and one participant submitting a partial survey response.

In Survey 2, one of the key aims was to develop consensus on using an ‘agreed umbrella term’ that broadly encompasses programs or services offered to people as they transition from custody into the community while using different ‘practice level’ terms at an individual/organisation level. Ninety-four percent of participants agreed that the use of an umbrella term was important, with the term ‘Transitional and Post-release Support Programs’ (TPSP) being ranked #1 by 54% of participants and the term ‘Community Transition and Post-release Programs’ (CIP) being ranked #2 by 37% participants. ‘Transitional Integration Programs’ (TIPs) was the least preferred term. Other questions related to best-practice principles, timing of the programs, responsibility for the funding, provision and coordination of programs, and monitoring.

At the end of this round, the Delphi study achieved consensus on all statements and the associated domains. See Table 2 for a summary of the proportion of statements where consensus was achieved.

**Table 2:** Proportion of best-practice statements (n = 40) where consensus was achieved.

Best-practice statement domains	Proportion of best-practice statements (n = 40) where consensus was achieved		
**Round 1, Survey 1** **(N = 84)**	**Round 1, Online meeting** **(N = 28)**	**Round 2, Survey 2** **(N = 35)**
**Definition & nomenclature**	5 out of 8 statements (62.5%)	100% consensus on introducing an agreed ‘umbrella term’ as part of ‘best-practice terminology’	–
**Best-practice terminology**	Revised term generated during the online meeting		Consensus on preferred term
**Aims & intended outcomes**	5 out of 5 statements (100%)	–	–
**Components & Attributes**	5 out of 7 statements (71.4%)	100% consensus to replace this theme with ‘best-practice principles’	–
**Best-practice principles**	Revised term generated during the online meeting		7 out of 8 statements (87.5%)
**Best-practice timing**	0 out of 4 statements (0.0%)	100% consensus on the importance of ‘timing of commencement & continuance’	3 out of 3 statements (100%)
**Responsibility**	3 out of 3 statements (100%)	–	–
**Monitoring & Evaluation**	12 out of 13 statements (92.3%)	–	2 out of 2 statements (100%)

The 40 statements that reached consensus were synthesised and collected under seven key themes to develop the final set of ‘best-practice statements’ for programs and services delivered to people transitioning from custody to the community. These key themes are:

i) Best-practice terminology and umbrella term
ii) Best-practice principles
iii) Aims and overarching goals for best-practice programs and services
iv) The three high priority/ essential interlinked stages of the custodial journey
v) Best-practice timing for commencement and continuation
vi) Primary responsibility for funding, provision and co-ordination
vii) Monitoring and evaluation

**Fig 2:**
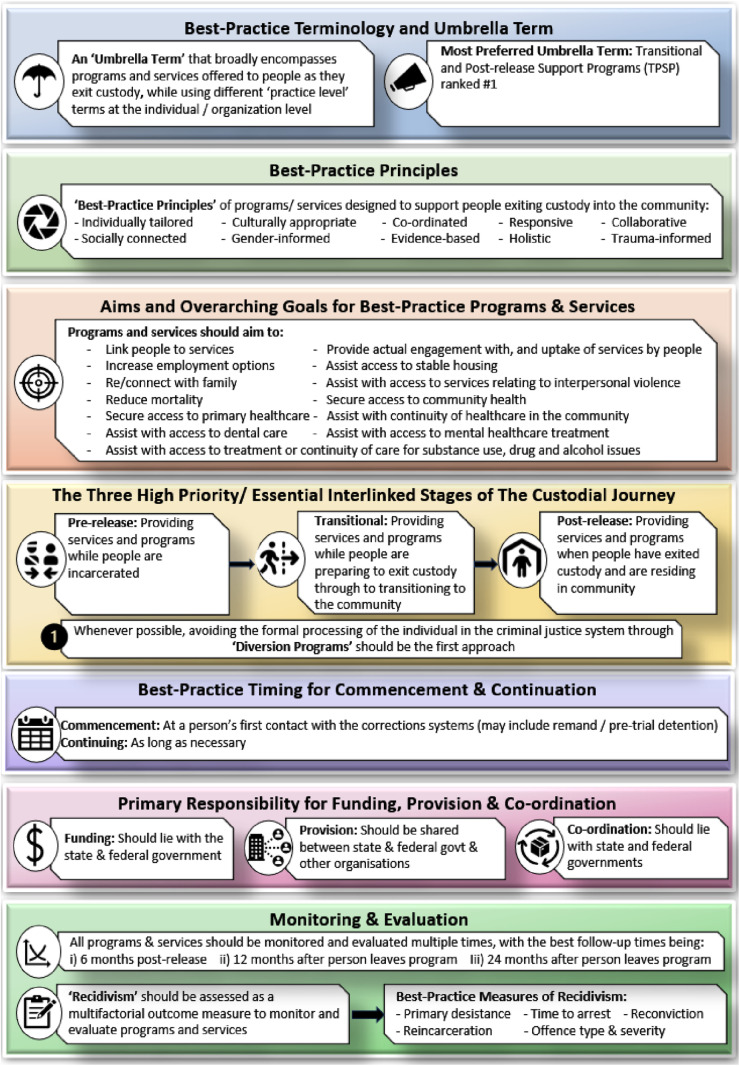
Best-practice statements on programs & services for people transitioning from custody to the community.

Details of the best-practice statements developed under each of these themes are presented in Fig 2.

In the context of existing universal basic principles for the treatment of people in custody [3], in particular, Rule number 4 of the Mandela Rules, which emphasizes the importance of creating favourable conditions for the reintegration of people who have been incarcerated into society under the best possible conditions [2]. These best-practice principles provide a consensus of essential elements of the Transitional and Post-release Support Program (TPSP), setting out what should be accepted as foundational in its design to support the reintegration of people exiting custody into the community.

In taking a human rights approach, it was also agreed that diversion programs should be the primary approach to criminal justice wherever local policing and the judicial environment support this. However, where deflection and diversion are not possible, interlinked transitional and post-release support programs are needed to provide continuity of care as people exit probation and transition into the community [40]. Our Delphi participants identified this approach as high priority/ essential.

**Fig 3:**
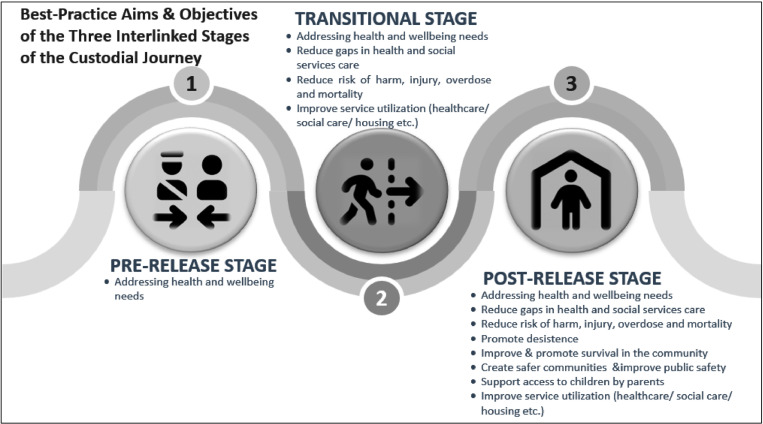
Aims and objectives associated with the best-practice model of the interlinked stages of programs and services offered to people exiting custody.

Where people are sentenced, any TPSP should be underpinned by a human rights informed program logic model with specific aims and goals, as detailed in Fig 3. For instance, depending on the program’s focus and its target population, objectives may include connecting people to services and increasing employment opportunities. Whereas, if the program is primarily targeting health, then one of its aims may be continuity of care that enables a smooth transition to accessing community health. This strategic approach will enable TPSP to effectively address the diverse needs of the population it serves, fostering successful reintegration and positive societal contributions.

There was also a very strong consensus throughout the process that all programs and services should be monitored and evaluated multiple times. The preferred follow-up times, as selected by participants, were 6 months post-release and 12 and 24 months after the person exits the program(s).

Results also indicated a strong consensus regarding the significance of implementing a seamless and continuous model of care and support for people exiting custody by using a staged approach for these programs and services. Specifically, there was 94% agreement for the implementation of a pre-release phase, 100% agreement that a Transitional phase, and 95.6% agreement that a post-release phase is ‘high priority’ or ‘essential’. Participants also indicated their consensus that specific best-practice aims and overarching goals are required for each of these stages (See Fig 3).

## Discussion

The United Nations Commission on Human Rights, (UNCHR) asserted that *‘Prisoners are the community. They come from the community, they return to it. Protection of prisoners is the protection of our communities’* [15]. Nevertheless, people returning to the community after imprisonment frequently encounter many obstacles in their efforts to reintegrate into society and exercise their fundamental rights in relation to housing, employment, health insurance coverage, education, or parenting.

Therefore, successfully exiting custody and surviving in the community remains a human rights problem for most people experiencing incarceration. There is no existing gold standard TPSP, further reflecting the complexity of providing person-centred support to people exiting custody and the heterogeneity of such programs. This complexity impacts both individuals receiving support and organisations providing these services. Consequently, this complexity precludes a one-size-fits-all approach to program design and service delivery.

Evidence-based best-practices should typically guide programs and services. However, in the absence of well-defined best-practice principles, achieving and measuring outcomes becomes challenging, limiting the capacity to benchmark and evaluate programs. This modified two-phased Delphi study delivers a set of best-practice statements that can be adapted to the individual needs and contexts of different programs and services globally. These statements clarify key themes such as language and terminology, program aims and outcomes, best-practice principles, the importance of an interconnected phased approach, the timing of delivery of services and programs, responsibility for funding and coordination of programs, and monitoring and evaluation. Rather than being prescriptive, these statements are intended for adaptation, interpretation and use for specific contexts by all involved stakeholders.

Adopting a unified language that bridges individual programs and services is crucial for achieving a humane, respectful, and coordinated response for people exiting custody. Using dignified, destigmatising, and humane terms are cornerstone of basic human rights and foster constructive engagement amongst service users and providers. The stigma attached to and enacted via labelling and using dehumanizing language fuels inherent biases against people who have been incarcerated. Consequently, these people are often socially excluded and denied economic resources and services that are essential for their survival in community. Through this study, we have successfully introduced a significant umbrella term, ‘Transitional and Post-Release Support Programs (TPSP),’ that has gained consensus among study participants. The implementation of agreed language and terminology and specifically this umbrella term TPSP, can enable cohesive efforts among government, public and private sector service providers, ultimately improving outcomes for people exiting custody. It also supports people to exit carceral settings with dignity in that it provides a framework to ensure that programs are guided by key principles, fostering constructive and respectful language. Recognising the significant variation in carceral policies and practices across different countries and continents, the use of TPSP as the agreed umbrella term also has the capacity to connect and engage national and international stakeholders and, as such, would likely result in funding proposals that are clearly linked and working toward common outcomes while allowing for specific best-practice principles and statements to be chosen for each program or service on a fit-for-purpose basis.

The best-practice principles (second box, Fig 2) generated from this Delphi process also serve as a valuable, evidence informed guide for various stakeholders, including people with lived experience, and other invested parties when designing, developing, and evaluating programs and services. For example, an intervention targeting young women in prison may use best-practice principles such as gender-informed and collaborative service principles. Further, a TPSP centred on skill development may incorporate one or more of these statements to design an individually tailored, transitional stage service that delivers culturally informed support and commences at the first point of contact with the criminal justice system. Other best-practice statements developed through this consensus-building project corroborate existing human rights calls such as prioritising deflection and diversion programs where possible [40]. This approach validates the United Nations Standard Minimum Rules for Non-custodial Measures (the Tokyo Rules) ‘*diversion programs should be the first approach wherever the local policing and judicial environment supports this’* (fourth box, Fig 2), calling on Member States to develop non-carceral measures by taking the human rights approach and provide other options to imprisonment, including rehabilitation needs of the offender [41].

A further benefit of using best-practice statements for TPSP relates to evaluation and monitoring. Few programs for people transitioning from custody into the community have been evaluated globally or in Australia [20,22]. These best-practice statements provide a consolidated research base and a shared understanding, helping service providers develop or refine initiatives and plan for program evaluation. Whenever possible, embedding the evaluation phase in the program design phase ensures the efficacy, effectiveness, efficiency, and sustainability of the TSPS.

**Fig 4:**
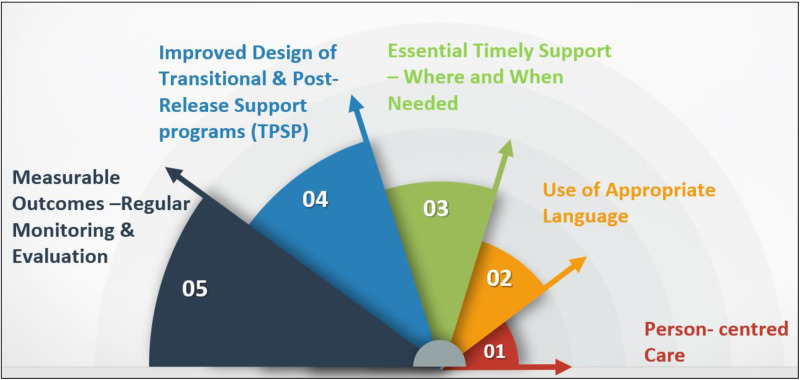
Value–Based TPSP: a strategic shift towards enhancing quality care and successful survival in community.

In summary, these best-practice statements for TPSPs focus on a value-based approach to enhance the quality of care for people exiting custody and improve their survival in the community, and although the consensus on these statements was primarily derived from Australian participants, these statements are designed to be easily adaptable for use across different global contexts (see Fig 4).

Overall, the results of this modified Delphi study present three key opportunities:

1. To adopt a common language for labelling, classifying, monitoring and reporting Transitional and Post Release Support Programs (TPSP) to destigmatise language that may otherwise negatively impact people exiting custody. Furthermore, an internationally consistent approach to using standardized terminology would lead to a common understanding between all stakeholders and funders and enable a more efficient transfer of knowledge and innovation.
2. To develop a framework for the evidence informed design, monitoring and evaluation of TPSPs, enabling national and international benchmarking of programs.
3. To develop, co-design, and offer efficacious, effective, and efficient programs based on human rights approaches to enhance people’s outcomes and survival in the community.

### Strengths and limitations

One main challenge of any consensus-based study is that the responses can be substantially influenced by the participants on the panel, rating scale and consensus criteria. For instance, the proportion of the study sample that included individuals with lived experience of incarceration and re-entry was relatively small. This limitation may affect the generalizability of the findings. However, the Delphi consensus-building process enabled open discussions during online meetings with participants, including people with lived experiences of incarceration and transition to the community, ensuring the authenticity and strength of consensus. Participation of the latter group in this Delphi process, in part, mitigated the limitation of not including people currently in custody in the process. Future research should explicitly include and prioritize the voices of people with lived experience to enhance the robustness of research and to lead and shape these programs. By doing so, the research can better capture the nuanced challenges and needs of this population, leading to more effective and impactful re-entry strategies. Incorporating their perspectives can also enhance the credibility and relevance of the findings, ensuring that the programs developed are truly reflective of the experiences and requirements of those they aim to support. While we were successful in securing the input of a small number of international participants, the majority of the participants were from Australia. Therefore, the findings of this study are primarily informed by the countries represented by the authors and study participants. We do not assume that these views represent the views and practices of different correctional facilities across the world. Hence, there is a need for expanding the dialogue to include a broader range of national and international participants, including people with lived experiences across different ethnicities, cultures and communities, so as to consider the diverse carceral policies and practices globally and to better understand how these differences impact re-entry services and outcomes.

However, one of the key strengths of this study is the development of best-practice statements that, despite the wide variation in carceral settings globally, can serve as guiding principles across all contexts. These statements are designed to be adaptable and interpretable for specific local conditions, providing a flexible framework that supports the development and refinement of TPSPs globally. Lastly, in alignment with the consensus reached by participants, this study exclusively focussed on recidivism as the primary outcome of interest. While recidivism is a critical measure of community reintegration, we acknowledge that other indicators of success, such as employment, housing stability, and social reintegration, are equally important for a holistic assessment of re-entry programs. Future research should incorporate these additional measures to provide a more comprehensive evaluation of re-entry outcomes.

## Conclusion

This study successfully developed the agreed umbrella term to describe programs and services for people exiting custody, Transitional and Post-Release Support Programs (TPSP), that is acceptable to a range of participants, including people with lived experience. This term should be adopted as a common language that bridges individual programs and services and enables effective communication across sectors using humane and destigmatized language. The consensus-building phase also resulted in the development of best-practice statements for TPSPs that can be used as a guideline for government, non-government agencies, people with lived experience and other stakeholders, and offer a foundational evidence-base for use in TPSP program design and service delivery. These statements provide guidance regarding what content should be included in diverse and complex programs, how these programs should be implemented, and implications for practice. Embedding best practices in programs and services can improve processes related to access, documentation, monitoring and evaluation. In conclusion, adopting the umbrella term TPSP will encourage the use of humane, dignified language in relation to programs for people exiting custody, while embracing best-practice principles and statements in relation to TPSPs will inspire a streamlined, timely, structured and collaborative discourse to purposive program design and a more cohesive approach to service provision for people exiting custody.

## Supporting information

Appendix 1 Figure a, Figure b. (ZIP)

S1 & S2 Round 1, Survey 1 Questionnaire and Round 2, Survey 2 Questionnaire. (PDF)

S3 Accord checklist. (PDF)
