# Supplementary figures and images for: Establishing best-practice statements for post-custody community transition: Insights from a modified Delphi study
Source: PLoS One. 2025 May 8;20(5):e0323118. doi: 10.1371/journal.pone.0323118 (PMC12061168; doi:10.1371/journal.pone.0323118)

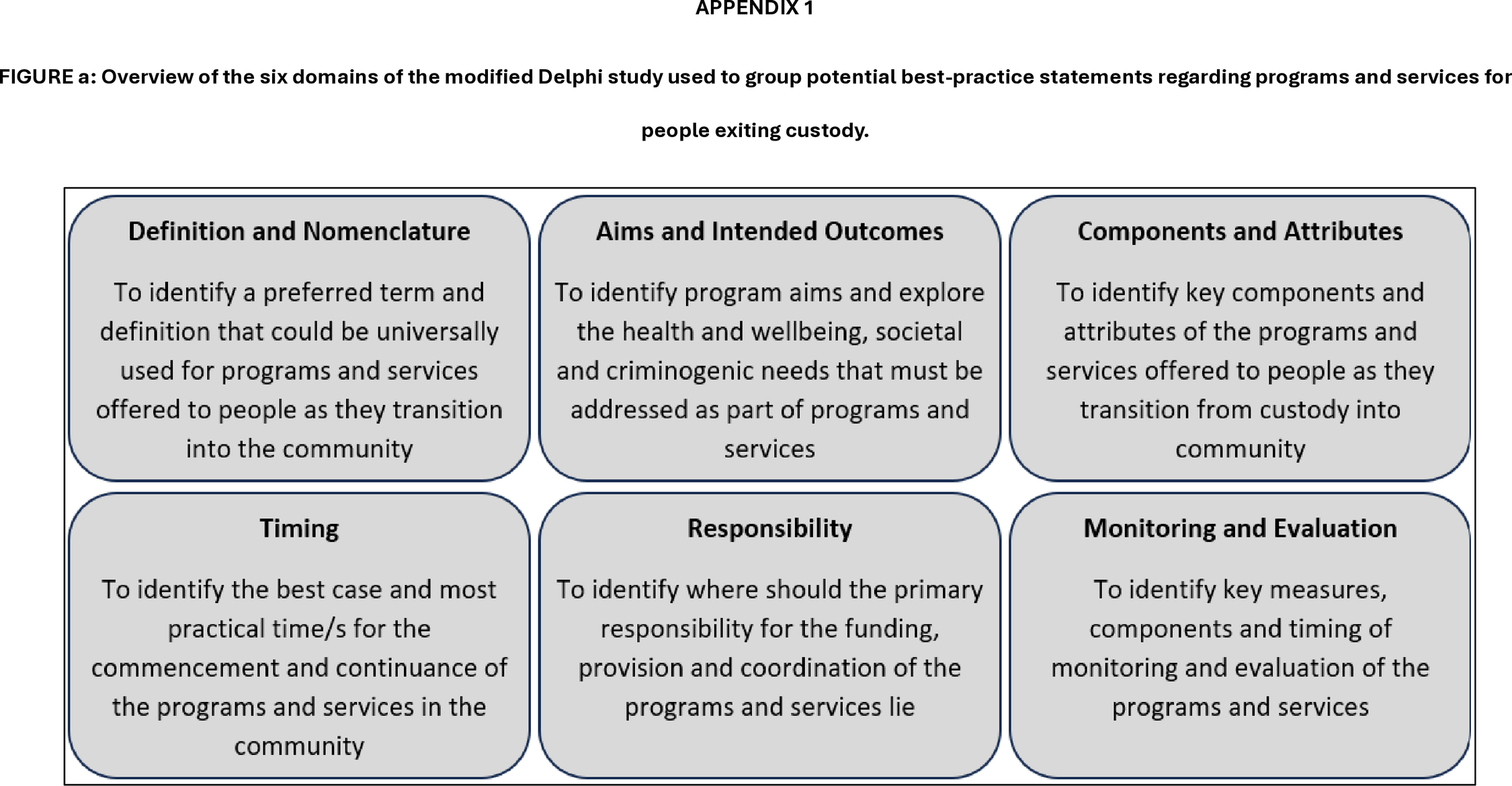

Supplement: Appendix 1 — (ZIP) [file pone.0323118.s001.zip › Appendix 1 (Figure a, b)/Appendix 1 (Figure a).tif]

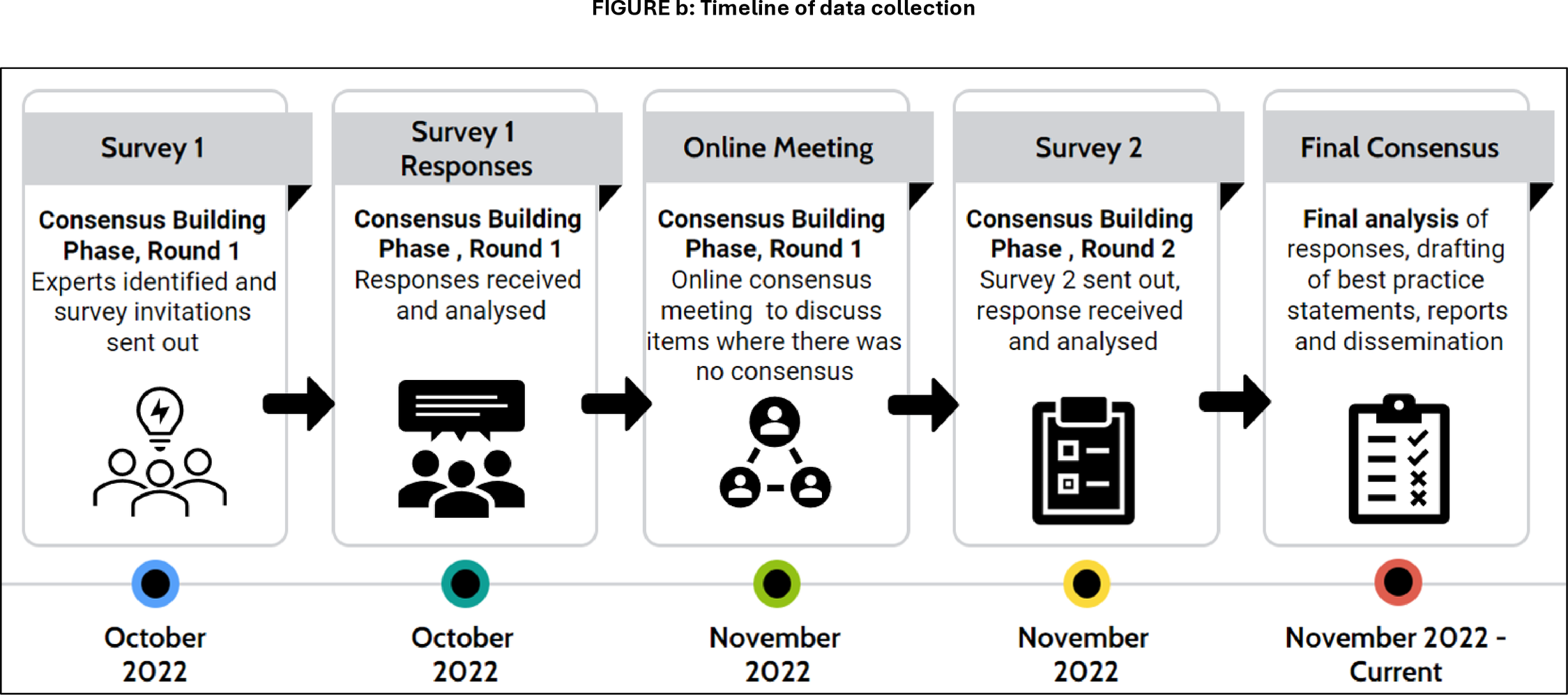

Supplement: Appendix 1 — (ZIP) [file pone.0323118.s001.zip › Appendix 1 (Figure a, b)/Appendix 1 (Figure b).tif]
